# The curvilinear relationship between work pressure and momentary task performance: the role of state and trait core self-evaluations

**DOI:** 10.3389/fpsyg.2015.01680

**Published:** 2015-10-28

**Authors:** Joeri Hofmans, Jonas Debusscher, Edina Dóci, Andromachi Spanouli, Filip De Fruyt

**Affiliations:** ^1^Department of Psychology, Work and Organizational Psychology, Vrije Universiteit Brussel, Brussel, Belgium; ^2^Department of Developmental, Personality and Social Psychology, Ghent University, Ghent, Belgium

**Keywords:** core self-evaluations, task performance, state, trait, within-person, between-person

## Abstract

Whereas several studies have demonstrated that core self-evaluations (CSE)–or one’s appraisals about one’s own self-worth, capabilities, and competences–relate to job outcomes, less is known about the mechanisms underlying these relationships. In the present study, we address this issue by examining the role of within- and between-person variation in CSE in the relationship between work pressure and task performance. We hypothesized that (a) work pressure relates to task performance in a curvilinear way, (b) state CSE mediates the curvilinear relationship between work pressure and task performance, and (c) the relationship between work pressure and state CSE is moderated by trait CSE. Our hypotheses were tested via a 10-day daily diary study with 55 employees in which trait CSE was measured at baseline, while work pressure, task performance, and state CSE were assessed on a daily basis. Bayesian multilevel path analysis showed that work pressure affects task performance via state CSE, with state CSE increasing as long as the employee feels that (s)he is able to handle the work pressure, while it decreases when the level of work pressure exceeds the employees’ coping abilities. Moreover, we found that for people low on trait CSE, the depleting effect of work pressure via state CSE happens for low levels of work pressure, while for people high in trait CSE the depleting effect is located at high levels of work pressure. Together, our findings suggest that the impact of work pressure on task performance is driven by a complex interplay of between- and within-person differences in CSE.

## Introduction

Most studies on the role of personality in work and organizational settings have focused on the Big Five dimensions, arguing that they cover a large part of what is referred to as personality (Barrick and Mount, 1991; Barrick et al., 2001). Whereas this claim has indeed been supported by a bulk of empirical research, it has also become clear that the Big Five personality dimensions are not all encompassing, with one important example being that they “fail to capture chronic differences in how individuals evaluate themselves” (Kacmar et al., 2009, p. 1572). Owning to this, scholars have started to study traits that tap more into self-evaluations. One personality dimension that is particularly relevant in this respect and that is gaining more and more popularity in the work and organization domain is core self-evaluations (CSE)—or the appraisals a person makes about his/her own self-worth, capabilities, and competences (Judge et al., 1998).

core self-evaluations is a broad personality dimension consisting of four lower-order dimensions: self-esteem (i.e., the worthiness that is attributed to oneself as a person); generalized self-efficacy (i.e., one’s beliefs about his/her ability to handle situations and solve problems); locus of control (i.e., one’s beliefs regarding his/her capacity to influence life’s events); and neuroticism (i.e., one’s inclination to focus on negative aspects of the self and experience negative affect; Judge et al., 2003). The validity and importance of CSE for the work and organizational domain has been supported by studies that demonstrated its predictive validity over and beyond each of the four separate CSE sub-dimensions (Erez and Judge, 2001), and over and beyond each of the Big Five personality dimensions (Judge et al., 2008) for the prediction of important work outcomes such as performance and job satisfaction.

Although previous research has shown that there is a relationship between stable, between-person differences in CSE and stable, between-person differences in job outcomes, no studies have focused on *if* and *how* CSE relates to job outcomes on a day-to-day basis. Nevertheless, such an understanding is important, both from a theoretical and a practical point of view. Theoretically, shifting the attention from between- to within-person fluctuations implies that CSE is no longer conceptualized as fixed, but rather as something that dynamically fluctuates as a function of everyday experiences. Hence, it becomes important to not only study the consequences of CSE, but also its day-to-day antecedents; an endeavor that will significantly increase our understanding of the mechanisms underlying the elicitation and functioning of CSE at work. On a practical level, conceptualizing CSE as a construct that is subject to within-person variation might open the door for job (re)design that takes into account these within-individual fluctuations or for various types of managerial interventions aimed at increasing employee CSE. In the present paper, we aim to expand our understanding of the mechanisms underlying day-to-day fluctuations in CSE by examining (a) how day-to day variation in work pressure is related to day-to day variation in CSE, (b) how variable, within-person differences in CSE dynamically interact with stable, between-person differences in CSE, and (c) how within- and between-person differences in CSE together relate to job performance.

### Within-person Fluctuations in CSE

Although CSE has traditionally been conceptualized as a stable personality trait (Judge et al., 1998), recent research indicates that it not only varies between but also within individuals (Schinkel et al., 2004; Debusscher et al., 2015b; Dóci and Hofmans, 2015). This is not surprising as self-efficacy, self-esteem, and neuroticism—all being sub-dimensions of CSE– have been shown to consist of a stable, between- as well as a variable, within-person component (Heatherton and Polivy, 1991; Bandura, 2006; McNiel and Fleeson, 2006; Debusscher et al., 2014, 2015a). Thus, even though individuals are inclined to habitually view themselves in a more positive or negative light, recent research suggests that their self-evaluations vary across time and in different circumstances (Judge and Kammeyer-Mueller, 2004); an idea that closely aligns with the new framing in personality psychology that focuses not only on between-, but also on within-person fluctuations (Fleeson, 2001; Funder, 2009). In line with this, the present study aims to reconcile the stable trait and the variable state perspectives by examining how state and trait CSE dynamically interact in daily working life.

To do so, we start from the Core Self-evaluations Job Affect Multilevel (CSEJAM) model of Judge et al. (2012). According to this model, variation in one’s work and life environment trigger variation in state CSE, which in turn relates to job affects and affect-driven behaviors. Turning to the interplay between trait and state CSE, Judge et al. (2012), in their CSEJAM model, argue that trait CSE moderates the relationship between the situational triggers and state CSE because it influences the extent to which the work and life environment trigger increases or decreases in state CSE. In other words, the CSEJAM model conceptualizes trait CSE as individual differences in the sensitivity to CSE-relevant situational provocation; a conceptualization that is also adopted in well-known person-situation interactionism models such as Trait Activation Theory (Tett and Guterman, 2000) and the Traits as Situational Sensitivities Model (Marshall and Brown, 2006).

In the present study, we draw on the CSEJAM model to study the relationships between work pressure, trait and state CSE, and task performance. The reason for focusing on work pressure as an antecedent and task performance as an outcome of state CSE is threefold. First, work pressure and task performance are everyday constituents of working life (Minbashian et al., 2010). Second, they are elements of all working environments, and therefore they generalize across tasks and situations. Third, research shows that a stressful working environment relates to correlates of CSE, such as stress, anxiety (Wood et al., 2011), self-efficacy, hope, optimism, and resiliency (Newman et al., 2014), while CSE (Chang et al., 2012) as well as its different subdimensions (Judge and Bono, 2001) has been shown to relate to task performance. In what follows, we will first discuss the within-person relationships between work pressure, state CSE, and task performance, and subsequently, we will discuss the moderating effect of trait CSE.

### Relating Work Pressure to Task Performance: The Mediating Role of State CSE

Recent research suggests that not all job demands are alike and that it is important to distinguish between hindrance and challenge demands (LePine et al., 2005). Hindrance demands, such as role conflict or red tape, are typically perceived as opposing personal growth and achievement, which implies that, even if employees are able to overcome them, they offer little to no potential gain (Cavanaugh et al., 2000). Instead, challenge demands such as task complexity and work pressure are perceived by employees as opportunities to learn and achieve, and therefore they create an opportunity for personal growth and goal achievement (Cavanaugh et al., 2000). However, besides their motivational effect, challenge demands are also energy-draining, manifested in the positive relationship with psychological strain and ill health (Boswell et al., 2004; LePine et al., 2004, 2005; Podsakoff et al., 2007).

The theory and empirical research on challenge demands suggest that work pressure has the potential to stimulate as well as deplete work outcomes; a dual function that is supported by an inverted U-shaped relationship between challenge demands on one hand and performance, motivation, job satisfaction, and other important work outcomes on the other hand (Xie and Johns, 1995; De Jonge and Schaufeli, 1998; Zivnuska et al., 2002). To explain this curvilinear relationship, researchers often draw on the Yerkes-Dodson law (Yerkes and Dodson, 1908) and activation theory (Gardner, 1986; Gardner and Cummings, 1988). Both theories suggest that at very low levels of activation, people are apathetic. Therefore, increases in work-related stimulation have an energizing effect when the current stimulation level is low. However, when the activation level is already high, increasing the level of work pressure further, might trigger the individual’s feeling that s/he can no longer cope with the high demands (Boswell et al., 2004; Webster et al., 2010), and under these conditions, performance, motivation, and other work-related outcomes start to deplete. Therefore, drawing on the Yerkes-Dodson law (Yerkes and Dodson, 1908) and activation theory (Gardner, 1986; Gardner and Cummings, 1988), we expect within-person variation in work pressure to relate to within-person variation in task performance in an inverted U-shaped way.

Hypothesis 1: Work pressure has an inverted U-shaped within-person relationship with task performance.

As mentioned above, work pressure has an energizing effect as long as the individual feels that s/he is able to cope with the demands at hand, while it becomes counterproductive if the level of work pressure exceeds the individual’s coping abilities (Boswell et al., 2004; Webster et al., 2010). Because state CSE reflects the momentary appraisals a person makes about his/her own self-worth, capabilities, and competences to cope with the environmental demands (Judge et al., 1998), we expect variation in perceived work pressure to trigger variation in state CSE. In particular, when working under little work pressure, people may feel in control, but at the same time they might feel under-stimulated, frustrated, and passive (Gardner, 1986; Gardner and Cummings, 1988; Zivnuska et al., 2002). As a result of this mixture of experiences, their state CSE will be sub-optimal. Instead, when experiencing a level of work pressure that is demanding but feasible “*people are likely to believe that there is a positive relationship between efforts expended on coping with these demands, and also likely to believe that if these demands are met, valued outcomes will occur.*” (LePine et al., 2005, p. 765). Under these conditions the person’s sense of self-esteem (Rodell and Judge, 2009), self-efficacy, and control is enhanced because of the perceived relationship between efforts and results, while positive emotions are triggered (LePine et al., 2005) because the person expects to obtain valued outcomes. This mixture of ingredients (i.e., high self-esteem, self-efficacy, and control, combined with low negative emotions or low state neuroticism) represents an optimal level of state CSE. Finally, when work pressure grows further it might become overwhelming, there by depleting the sense of self-efficacy and self-worth, evoking the feeling that the person is no longer in control, and boosting state neuroticism because of increased feelings of anxiety (Zivnuska et al., 2002). In other words, when job demands become excessive, they exhaust one’s personal resources—which in this study are captured by CSE– (Bakker et al., 2003, 2004). This idea of a curvilinear relationship between job demands and how one acts, feels, and thinks has been supported by research showing that challenge stressors relate curvilinearly to anxiety and emotional exhaustion (Xie and Johns, 1995; De Jonge and Schaufeli, 1998). In summary, we suggest that the relationship between work pressure and state CSE is inverted U-shaped; it peaks at moderate levels and declines at low and high levels of work pressure.

*Hypothesis 2: Work pressure has an inverted U-shaped within-person relationship with state CSE*.

In the foregoing, we have argued that within-person variation in work pressure triggers within-person variation in CSE, and that performance varies as a function of the extent to which the individual feels that s/he can cope with the situational demands, which in the present study is captured by the level of state CSE. Although there is to the best of our knowledge only one within-person study on the positive relationship between CSE and task performance (Debusscher et al., 2015b), meta-analytical research has shown that, at the between-person level, CSE (Chang et al., 2012) as well as its four sub-dimensions (Judge and Bono, 2001) is positively related to task performance. An important reason for the positive relationship between CSE and task performance is that individuals who are high on CSE are better at setting goals, working toward them, and are as a result more motivated to perform their jobs. Indeed, both in a lab experiment and a field study, Erez and Judge (2001) demonstrated that CSE related to task motivation, persistence, goal setting, goals commitment, activity level, and task performance. Building on these findings, we hypothesize that day-to day variation in state CSE relates positively to day-to day variation in task performance, which, when combined with the foregoing hypotheses, implies that state CSE is expected to mediate the curvilinear within-person relationship between work pressure and task performance.

*Hypothesis 3: State CSE mediates the inverted U-shaped within-person relationship between work pressure and task performance*.

### The Impact of Trait CSE on the Within-person Work Pressure-state CSE Relationship

Following the CSEJAM model (Judge et al., 2012) and person-situation interactionism models, we expect trait CSE to moderate the relationship between work pressure and state CSE. This expectation follows from the conceptualization of traits as individual differences in the sensitivity to situational provocation. Moreover, it relates to the concept of contingent units of personality, which represent the extent to which a single individual’s expression of a personality trait is contingent upon a specific feature of the situation (Minbashian et al., 2010). Building on the idea of traits as situational sensitivities, we argue that trait CSE relates to contingent units of CSE (i.e., the extent to which CSE is contingent upon work pressure).

In particular, and in line with Trait Activation Theory (Tett and Guterman, 2000) and the Traits as Situational Sensitivities Model (Marshall and Brown, 2006), we expect the within-person relationship between work pressure and state CSE to be weaker for people high on trait CSE than for people low on trait CSE. That is, for a person high in trait CSE, we expect the level of state CSE to be less contingent upon the level of work pressure because they are less susceptible to it. This reasoning is in line with the finding that people high in trait neuroticism react more strongly to negative environmental features than people low in neuroticism, even when confronted with relatively small problems (Suls and Martin, 2005; Debusscher et al., 2015a). In the same vein, Bolger and Schilling (1991) demonstrated that people high in trait neuroticism have an increased reactivity to stressful situations. Regarding self-efficacy, O’Connor et al. (2009) demonstrated that people with low trait self-efficacy are more susceptible to hassles than people high on trait self-efficacy. Finally, for self-esteem, it has been shown that people high in trait self-esteem are protected from the effects of external factors (Mossholder et al., 1981). As emotional stability (being the counterpart of neuroticism), high self-esteem, and high self-efficacy are indicators of high CSE, these findings suggest that people high in trait CSE might be less susceptible to variation in work pressure than low trait CSE people.

*Hypothesis 4: Trait CSE moderates the within-person relationship between work pressure and state CSE, such that the relationship is stronger in individuals with lower trait CSE*.

## Materials and Methods

### Participants

Fifty-five employees (33 women) from different Belgian companies participated in the study. On average, respondents were 44.31 years old (SD = 11.29) and their mean company tenure was 15.65 years (SD = 11.97). Fifteen participants had a secondary school degree, 12 completed a higher professional education, and 28 completed higher academic education. In terms of job content, 16 worked in logistics and distribution, 13 in governmental and non-profit organizations, 6 in health care, 6 in telecom, 4 in the financial sector, 1 in chemistry and pharmacy, 3 in human resources, 2 in communication, and 4 in other jobs. Ten participants worked part-time (seven participants worked 4 days, one participant worked 3 days, and two worked 2.5 days a week), and they only filled out the daily questionnaires on days on which work was done.

### Procedure

The study was approved by the Human Research Ethics Committee of the Vrije Universiteit Brussel (Dossier ECHW2015-17). We recruited participants in several ways. We posted a call on the intranet of the Flemish education networks, in the alumni newsletter of the Vrije Universiteit Brussel, and we emailed personal contacts. In these calls, we explained the goal of the study and stressed that the anonymity of records would be ensured. We only contacted people again who indicated that they were willing to participate in the study (via email or orally).

Participants were enrolled in a 10-day daily diary study in which trait CSE was measured at baseline, while work pressure, state CSE, and task performance were assessed daily. For the daily diary part, participants received an email each working day including a link to a survey in which they had to report on their level of work pressure, state CSE, and level of task performance, and they did so for 10 consecutive working days. At the beginning of each survey, we again stressed that the data would be made anonymous. Moreover, participants could stop participating in the study whenever they wanted. All scales, as well as the items within each scale, were randomized. Following this procedure, we collected 327 out of a maximum of 550 (55 employees × 10 days) data points, corresponding to a response rate of 59.45 percent.

### Measures

#### Trait CSE

Trait CSE was measured using the twelve-item CSE-scale of Judge et al. (2003). An example item of this scale is “Overall, I am satisfied with myself.” The items were rated on a seven-point scale, ranging from “completely disagree” to “completely agree.” The alpha reliability coefficient of this scale was 0.84.

#### State CSE

Because personality states can be defined as momentary enactments that have “*the same affective, behavioral, and cognitive content as their corresponding traits*” (Fleeson, 2012, p. 52), state CSE was also measured using the trait CSE-scale of Judge et al. (2003). To allow for a momentary or state measure of CSE, we slightly adapted the items (e.g., “Since this morning, I was satisfied with myself”). The items were rated on a seven-point scale, ranging from “completely disagree” to “completely agree.” To test the reliability of the scale, we used the multilevel confirmatory factor analysis approach of Geldhof et al. (2014), which revealed that the within-person omega reliability coefficient was 0.73.

#### Work Pressure

Work pressure was measured using the three-item scale of Bakker et al. (2003). Similar to the state CSE scale, we slightly adapted it to allow for daily ratings of work pressure (e.g., “Today, I had too much work to do”). All items had to be rated on a seven-point scale, ranging from “completely disagree” to “completely agree.” The within-person omega reliability coefficient was 0.80.

#### Task Performance

Task performance was measured using the seven-item task performance subscale of Williams and Anderson (1991). Similar to the state CSE scale, we slightly adapted it to allow for momentary self-ratings of performance (e.g., “Since this morning, I adequately completed assigned duties”). The seven items had to be rated on a seven-point scale, ranging from “completely disagree” to “completely agree.” The within-person omega reliability coefficient equaled 0.75.

### Analyses

Because of the complexity of the mediation model, we first tested all hypothesized relationships separately using two-level regression analyses with the lme4 package in R (Bates, 2010). All level-1 predictors (i.e., work pressure and state CSE) were person-centered, while the level-2 predictor (i.e., trait CSE) was grand-mean centered. This procedure ensures that the level-1 predictors contain within-person variability only, which is necessary because the hypotheses regarding the relationships between work pressure, state CSE, and task performance pertain to the within-person level. To test whether the effect of the level-1 predictors was consistent across individuals, we tested whether a model with a random slope on the between-person level fitted our data significantly better than a model without random slopes. Both models were compared using a log-likelihood difference test, and when the slope was non-significant (*p* > 0.05), it was trimmed.

Next, the hypotheses were tested simultaneously using Bayesian two-level path modeling in Mplus version 7.3 (Muthén and Muthén, 1998–2012). We used Bayesian estimation because it can flexibly accommodate non-normal distributions (Muthén, 2010; Kruschke et al., 2012; Zyphur and Oswald, 2013), which is important when testing for mediation using the product-of-coefficients approach (i.e., the product of two coefficients is traditionally non-normally distributed). Moreover, it allows testing complicated models. An important difference between Bayesian and the more traditional—frequentist—approach is that Bayesian analysis does not yield *p*-values and confidence intervals. Instead, for each parameter in the model, Bayesian analysis yields a posterior distribution, which shows the probability distribution of the parameter given the data (Kruschke et al., 2012; Zyphur and Oswald, 2013). Based on these posterior distributions, credibility intervals can be constructed. These credibility intervals include a predefined percentage of the posterior distribution (e.g., 95%), thereby returning the most credible values of the parameter. For our Bayesian analysis, we will draw on these credibility intervals to help deciding which parameter values should be deemed credible or not (Kruschke et al., 2012).

## Results

Means, standard deviations, correlations, and intra-class correlations (ICCs) of work pressure, state CSE, momentary task performance, and trait CSE are shown in Table 1. These ICCs show, for each level-1 variable, the proportion of variation due to between- and within-person differences. Overall, the ICCs show that a substantial part of the variability in work pressure, state CSE, and task performance is due to within-person differences.

**TABLE 1:** **Means, standard deviations, intra-class correlations and correlations for all study variables**.

****	**M**	**SD**	**ICC_between–person_**	**ICC_within–person_**	**1**	**2**	**3**
1. Work pressure	3.09	1.11	0.55	0.45			
2. State CSE	5.34	0.91	0.77	0.23	0.01		
3. Task performance	5.60	0.76	0.45	0.55	0.26**	0.33**	
4. Trait CSE	5.05	0.81	–	–	0.34*	0.77**	0.37**

**p < 0.01 (two-tailed); *p < 0.05 (two-tailed); M, mean; SD, standard deviation; ICC, intra-class correlation. The correlations between work pressure, state CSE, and task performance are within-person correlations (i.e., computed on person-centered data; N = 327). The correlations with trait CSE are between-person correlations (i.e., to compute them, work pressure, state CSE, and task performance were aggregated to the person-level; N = 55).

**FIGURE 1:**
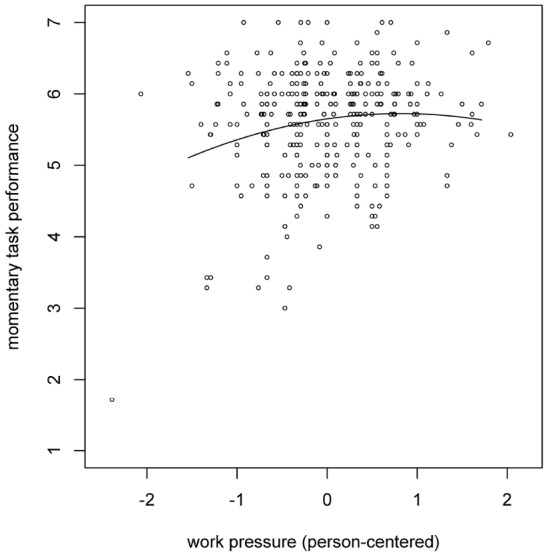
**Momentary task performance as a function of work pressure.** The work pressure scores are person-centered.

**FIGURE 2:**
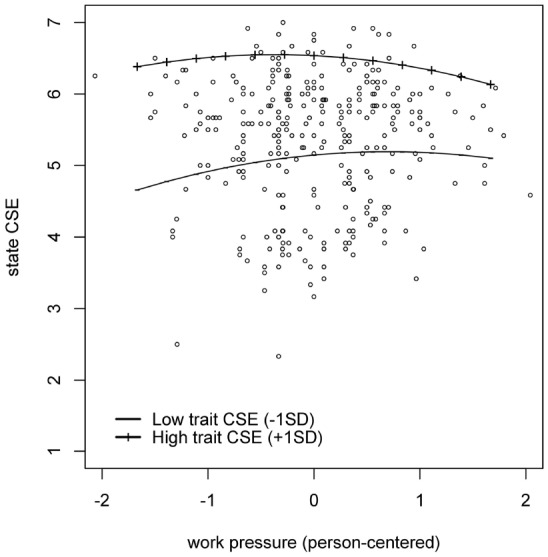
**State Core Self-Evaluations as a function of work pressure.** The work pressure scores are person-centered.

Next, we tested the hypothesized relationships by means of a series of two-level regression analyses. First, we tested whether within-person fluctuations in work pressure relate in an inverted U-shaped way to within-person fluctuations in task performance (i.e., Hypothesis 1). To do so, we predicted momentary task performance from work pressure and work pressure squared (work pressure was person-centered before computing the squared effect). Moreover, we tested whether these relationships varied across individuals. In line with Hypothesis 1, we found that both the linear (γ = 0.18; *p* = 0. 004) and the quadratic (γ = –0.11; *p* = 0. 041) component of work pressure related to momentary task performance (see Figure 1). Furthermore, the impact of both the linear (σ^2^ = 0.08; *p* < 0.001) and the quadratic (σ^2^ = 0.02; *p* = 0.003) component differed across individuals. Next, we tested whether there is an inverted U-shaped within-person relationship between work pressure and state CSE (i.e., Hypothesis 2). This analysis revealed that the quadratic (γ = –0.10; *p* = 0.015), but not the linear (γ = 0.03; *p* = 0.434) component of work pressure related to state CSE, thereby supporting Hypothesis 2. Moreover, we found between-person differences in the strength of the relationship between the linear component of work pressure and state CSE (σ^2^ = 0.02; *p* = 0.006), but not in the relationship between the quadratic component of work pressure and state CSE (σ^2^ = 0.01; *p* = 0.614). Thirdly, we tested the moderating effect of trait CSE on the relationship between work pressure and state CSE (i.e., Hypothesis 4). This was done by adding the main effect of trait CSE as well as the interaction between trait CSE and the linear component of work pressure to the previous model. In line with Hypothesis 4, this analysis showed that trait CSE negatively moderated the relationship between work pressure and state CSE (γ = –0.12; *p* = 0.024). Moreover, there was a positive direct effect of trait CSE on state CSE (γ = 0.80; *p* < 0.001). A graphical representation of this moderation effect is shown in Figure 2, which shows that the level of state CSE of people high on trait CSE is less affected by the level of work pressure these people experience. Finally, we tested a model in which momentary task performance was predicted by state CSE, work pressure, and work pressure squared^1^. This analysis showed that state CSE (γ = 0.36; *p* < 0.001) and work pressure (γ = 0.17; *p* = 0.004) related positively to momentary task performance, but work pressure squared did not (γ = –0.06; *p* = 0.210). Moreover, the impact of work pressure (σ^2^ = 0.07; *p* < 0.001) and work pressure squared (σ^2^ = 0.01; *p* = 0.019) differed across individuals, while this was not the case for state CSE (σ^2^ = 0.01; *p* = 0.241).

Next, we tested the moderated mediation model in its entirety using Bayesian two-level path analysis. To this end, a model was tested in which state CSE was predicted by the linear and squared effect of work pressure, while momentary task performance was predicted from state CSE and the linear and squared effect of work pressure (all these relationships were modeled at the within-person level). Moreover, and in line with the results of the multilevel regression analyses, we included random slopes for the relationship between work pressure and state CSE, the relationship between work pressure and momentary task performance, and the relationship between work pressure squared and momentary task performance. At the between-person level, the random slope between work pressure and state CSE was regressed on trait CSE^2^. To formally test the indirect (mediation) effect of work pressure on momentary task performance via state CSE (i.e., Hypothesis 3), we relied on the approach of Hayes and Preacher (2010), which is specifically developed for testing non-linear mediation. Because the relationship between work pressure (*X*) and state CSE (i.e., the *a*-path) is curvilinear, while the relationship between state CSE and momentary task performance (i.e., the *b*-path) is linear, the mediation effect not only depends on the *a*- and *b*-paths, but also on *X*, which implies that the effect of work pressure on momentary task performance via state CSE is conditional on the level of work pressure. Because of this reason, Hayes and Preacher (2010) refer to the indirect effect as the instantaneous indirect effect, which is the effect of the predictor on the outcome through the mediator(s) at a specific value of the predictor.

**FIGURE 3:**
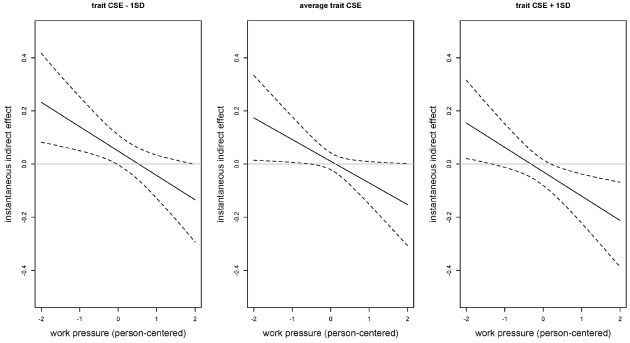
**The (instantaneous indirect) mediation effect of work pressure on momentary task performance via state core self-evaluations (CSE) as a function of work pressure (person-centered values).** The left panel shows the mediation effect for people scoring 1 SD below the average on trait CSE; the middle panel shows the mediation effect for people with an average level of trait CSE; and the right panel shows the mediation effect for people scoring 1 SD above the average on trait CSE. The work pressure scores are person-centered. The dotted lines represent the 95% credibility intervals.

A graphical representation of the instantaneous indirect effects, together with the 95% credibility intervals for people low (–1 SD), average, and high (+1 SD) on trait CSE is shown in Figure 3. From this figure, it can be seen that for low levels of work pressure the instantaneous indirect effect of work pressure on task performance via state CSE is positive [e.g., for people with a low (–1 SD) trait CSE score the instantaneous indirect effect equals 0.23 when the level of work pressure is low (i.e., a score of –2)]. This implies that, when work pressure is low, further increases in work pressure promote task performance via their effect on state CSE. Moreover, because the curves—describing the instantaneous indirect effect– decrease, the motivational effect of increases in work pressure weakens with increased levels of initial work pressure. On the contrary, for high initial levels of work pressure, the instantaneous indirect effect of work pressure on task performance via state CSE is negative [e.g., for people with a low (–1 SD) trait CSE score the instantaneous indirect effect equals –0.13 when the level of work pressure is high (i.e., a score of 2)]. This means that further increases in work pressure deplete task performance via their negative effect on state CSE. Moreover, this depleting effect becomes stronger when the initial level of work pressure is higher (which can be seen from the fact that the curves decrease). Combined, Figure 3 thus provides support for a curvilinear mediation effect (i.e., Hypothesis 3) as increases in work pressure are promoting task performance via state CSE when the level of work pressure is low, while they deplete task performance via state CSE when the level of work pressure is high. Regarding the moderation effect of trait CSE, Figure 3 shows that for people low in trait CSE the depleting effect of work pressure via state CSE especially holds for low levels of work pressure, while for people high in trait CSE the depleting effect is especially located at high levels of work pressure. This can be seen from the fact that the curves shift downward when going from low to high trait CSE, and from the fact that the 95% credibility intervals contain 0 at high (respective low) values of work pressure for people low (respective high) in trait CSE.

## Discussion

With the present paper, we contributed to a better understanding of the role of CSE at the workplace. This was done by (a) shedding light on a work-related trigger (i.e., perceived work pressure) and consequence (i.e., task performance) of state CSE and (b) by revealing the unique way in which state and trait CSE interact. This is a major contribution to the literature on CSE, as it uncovers the mechanisms through which CSE relates to work outcomes in everyday working life. In what follows, we will discuss the theoretical and practical implications of our findings.

### Theoretical Implications

In line with the CSEJAM model (Judge et al., 2012) and person-situation interactionism models (Tett and Guterman, 2000; Marshall and Brown, 2006), we found that trait CSE can be conceived of as individual differences in the extent to which appraisals about one’s sense of self-worth, capabilities, and competence depend on environmental stimulation. This showed from the fact that the relationship between work pressure and state CSE differed as a function of the individual’s level of trait CSE.

Importantly, our findings not only support, but go well beyond the mechanisms proposed by person-situation interactionist models such as Trait Activation Theory (Tett and Guterman, 2000) and the Traits as Situational Sensitivities Model (Marshall and Brown, 2006) by showing that the mediation effect of work pressure on task performance via state CSE is not only quantitatively, but also qualitatively different for people with different levels of trait CSE. That is, for people low in trait CSE, the depleting effect of work pressure via state CSE operates for low but not for high levels of work pressure, while for people high in trait CSE the depleting effect is located at high but not at low levels of work pressure. Altogether, this suggests that, depending on one’s trait CSE level, qualitatively different mechanisms might be at play.

We suggest that this dual mechanism can be explained by goal setting (Locke and Latham, 1990) and self-discrepancy theory (Higgins, 1987). In particular, low levels of work pressure might not pose a problem for people high in trait CSE because these individuals have a higher level of goal setting motivation (Erez and Judge, 2001). An important reason for this might be that goal commitment—which is an element of goal setting motivation– is a function of expected goal attainment, and this is per definition higher in people who are high in trait CSE. Because people high in trait CSE have higher levels of goal commitment, they do not require external pressure to perform well. People low in trait CSE, in contrast, do not have this strong base of resources, and therefore rely more on external pressures to regulate their behavior. Indeed, because they are less likely to believe that they can achieve what they want to achieve, their level of goal commitment is generally lower. Therefore, their level of state CSE is more strongly influenced by external pressures when the level of work pressure is low. The result of all of this is that under conditions of low work pressure, the level of state CSE of high trait CSE people is virtually unaffected when work pressure decreases, while the level of state CSE of low trait CSE people decreases because of the combination of under-stimulation and a lack of goal setting motivation. Turning to high levels of work pressure, we believe that the reason for the detrimental effect of increased levels of work pressure on the state CSE of individuals high on trait CSE, may be that their self-image strongly relies on the idea that they succeed in whatever they undertake. However, when they come across a situation in which the level of work pressure is (too) high, this high sense of achievement gets threatened, which, according to self-discrepancy theory (Higgins, 1987), leads to a flow of negative emotions such as disappointment, dissatisfaction, sadness, and depression. People with a low trait CSE level, in turn, should experience these feelings of self-discrepancy to a lesser extent because for them not being able to cope with the demands at hand is nothing new, and is more congruent with their self-image. As a result, under high work pressure, the level of state CSE of people high on trait CSE decreases when high work pressure increases further due to increasing feelings of self-discrepancy, while the state CSE level of people low on trait CSE does not decrease substantially because being unable to meet demands is not perceived as a shock for their self-image. It should be noted that, to formally test this dual mechanisms account, future research is needed in which goal commitment and self-discrepancy are measured along with work pressure, state CSE, trait CSE and task performance.

A possible alternative explanation for the finding that there are qualitatively different mediation effects for people high and low on trait CSE is that the levels of perceived work pressure might not be comparable. Because we person-centered the perceived work pressure scores, all between-person differences in work pressure were removed from the data. Yet, it might be that that the baseline of work pressure is higher for trait CSE people, as they seek and create jobs that offer challenges; an idea that aligns with the finding that people select situations that are congruent with their personality (Emmons et al., 1986; Côté and Moskowitz, 1998; Frederickx and Hofmans, 2014). Because of this (which is by the way supported by the positive correlation between the person’s average level of work pressure and his/her trait CSE level), people high on trait CSE might experience generally higher levels of work pressure than people low on trait CSE. As a result, for people high on trait CSE, levels of work pressure that are lower than usual can still be relatively high, and therefore they might still be challenging and not be associated with apathy. Conversely, levels of work pressure higher than usual might be extremely high for people high in trait CSE, which would then lead to overload and depletion of their state CSE level. For people low in trait CSE, levels of work pressure lower than usual may be very low and therefore offer no stimulation at all, hence depleting their state CSE level. When experiencing more work pressure than usual, the level of work pressure might be high, but still manageable for those low in trait CSE; and therefore it should not relate to decreased levels of state CSE. We tested this alternative explanation by regressing state CSE on the grand-mean centered work pressure scores (which contain both between- and within-person variability). Although the effects are weaker (i.e., the effects of work pressure squared and the interaction between trait CSE and work pressure are only significant at the *p* < 0.10 level), the pattern of findings was similar to that found with person-centered scores. This implies that between-person differences in work pressure cannot fully explain the qualitatively different mechanisms. However, to find a definite answer to the question whether individual differences in the average level of work pressure might explain why people with different trait CSE levels react differently to work pressure, future research is needed. One way to do so would be to manipulate work pressure rather than to measure it.

### Practical Implications

In line with previous findings on challenge demands, our study shows that, up to some point, work pressure might stimulate state CSE and task performance. This implies that managers should not always try to decrease the level of work pressure. Instead, they might try to keep work pressure at a moderate level as this seems to work best with all employees. Additionally, our findings also revealed that the mechanism relating work pressure to task performance is different for people with different trait CSE levels. While increasing low levels of work pressure can activate people low on trait CSE because it increases their state CSE level, it has little effect on people high on trait CSE. This implies that managing the level of work pressure is especially relevant when the employee is low in trait CSE, as for these people increasing challenge demands can trigger resources. Finally, very high levels of work pressure should always be avoided as they strongly deplete the state CSE of people high, and do no longer activate the state CSE of people low in trait CSE.

### Limitations and Future Research

Despite its strong points, our study is also subject to some limitations. First, all data were self-reported and came from a single source. Whereas self-reports are needed to measure CSE, they might be problematic for work pressure and task performance because of self-serving biases. Yet, because of the way we centered the data (i.e., relative to the individual’s own baseline), consistent over- or underestimations of the level of work pressure and task performance are absorbed by the individual’s average and are therefore removed from the data. As a result, stable, between-person differences in self-serving biases cannot account for our findings. However, when the degree of over- or underestimation varies as a function of one’s level of state CSE, this cannot be resolved with person-centering the data. To solve this issue, one should rely on other-rated work pressure and task performance and/or on objective measures of these variables. Note, however, that collecting other-ratings might be challenging in a daily diary study as peers or supervisors typically do not monitor one’s task performance on a day-to-day basis. Objective task performance, on the other hand, may resolve the issue of self-serving bias, but introduces external validity issues as objective task performance can only be collected for a very limited number of occupational groups.

A second limitation is that the data are correlational in nature. This implies that we were able to show that work pressure, state CSE, and task performance were related at the within-person level, but not that work pressure caused state CSE, and that state CSE in turn led to task performance. To test such causal relationships, experimental research is needed.

### Conclusion

Our findings suggest that the impact of work pressure on task performance is driven by a complex interplay of between- and within-person differences in CSE. Regarding this interplay, we supported and extended the idea of traits as individual differences in the susceptibility to situational provocation by (a) showing that trait CSE predicts how people react to within-person fluctuations in work pressure, and (b) that this differential reactivity is qualitatively different for people low and high in trait CSE. These findings have important implications for future research and practice because they suggest that different mechanisms are at play.

### Conflict of Interest Statement

The authors declare that the research was conducted in the absence of any commercial or financial relationships that could be construed as a potential conflict of interest.

## Footnotes

1 Although this test does not directly addresses one of our hypotheses, we performed it to decide whether or not to include random slopes for the direct effects (i.e., the relationships between work pressure and work pressure squared on one hand and momentary task performance on the other hand) in the Bayesian path model.

2 The results from the Bayesian path analysis fully replicated the findings of the separate regression analyses.
